# Acute necrotizing colitis with cecal perforation in a patient with autosomal dominant polycystic kidney disease: a case report

**DOI:** 10.1093/jscr/rjaf594

**Published:** 2025-08-06

**Authors:** Yahya Almarhabi

**Affiliations:** Center of Excellence in Trauma and Accidents, King Abdulaziz University, Jeddah 21589, Saudi Arabia; Department of Surgery, Faculty of Medicine, King Abdulaziz University, Jeddah 21589, Saudi Arabia

**Keywords:** autosomal dominant polycystic kidney disease (ADPKD), necrotizing colitis, cecal perforation, dialysis complications, surgical case report, gastrointestinal perforation

## Abstract

Autosomal dominant polycystic kidney disease (ADPKD) is a common hereditary disorder with both renal and extrarenal manifestations. While hepatic cysts and intracranial aneurysms are well-known complications, gastrointestinal involvement is rarely reported. Colonic perforation and necrotizing colitis in ADPKD patients, especially those undergoing dialysis, represent an unusual and serious clinical scenario. We report a rare case of acute necrotizing colitis with cecal perforation in a 50-year-old female with a known history of ADPKD, hypertension, and end-stage renal disease on dialysis. The patient presented with vague abdominal pain, systemic signs of sepsis, and was found to have free intraperitoneal air. Exploratory laparotomy revealed cecal perforation with necrotic segments, prompting ileocecal resection. Histopathological examination confirmed acute necrotizing colitis with two perforated ulcers. The case underscores the importance of early recognition and intervention in ADPKD patients presenting with acute abdomen, highlighting an uncommon but life-threatening complication.

## Introduction

Autosomal dominant polycystic kidney disease (ADPKD) is a hereditary disorder marked by progressive renal cyst development and extrarenal manifestations. It is the most common hereditary kidney disease, affecting ⁓1 in 400–1000 live births and accounting for 5%–10% of end-stage renal disease cases worldwide [1]. The condition arises from mutations in the PKD1 or PKD2 genes, leading to the formation of numerous renal cysts and the gradual loss of kidney function.

In addition to renal manifestations, ADPKD is associated with extrarenal complications, including hepatic cysts, intracranial aneurysms, cardiac valve abnormalities, and gastrointestinal involvement. Liver cysts are the most frequent extrarenal feature, present in up to 80% of affected individuals by the age of 60 [2]. Colonic involvement, while less common, may include diverticulosis and an increased risk of bowel perforation, particularly in patients undergoing dialysis [3].

Gastrointestinal complications such as acute necrotizing colitis and colonic perforation are rare and underreported. These complications can be exacerbated by the immunosuppressive state of uremia, altered colonic motility, and vascular compromise due to hypotension or anemia, which are frequently encountered in end-stage renal disease [4]. This report highlights a rare case of necrotizing colitis with cecal perforation in a dialysis-dependent ADPKD patient.

## Case presentation

A 50-year-old female with known ADPKD, essential hypertension, and end-stage renal disease requiring hemodialysis presented to the emergency room with a 3-day history of diffuse abdominal pain, anorexia, vomiting, and fatigue. She had two brothers also diagnosed with ADPKD on dialysis.

On admission, her vital signs were: heart rate 108 bpm, blood pressure 102/60 mmHg, temperature of 38.6°C, and Glasgow Coma Scale of 12. Abdominal exam revealed lower abdominal tenderness, generalized guarding, and signs of peritonitis. Labs showed Hb 6.9 g/dL, platelets 97 000/μL, C-reactive protein 26.6 mg/L, and metabolic acidosis (pH 7.12).

**Figure 1:**
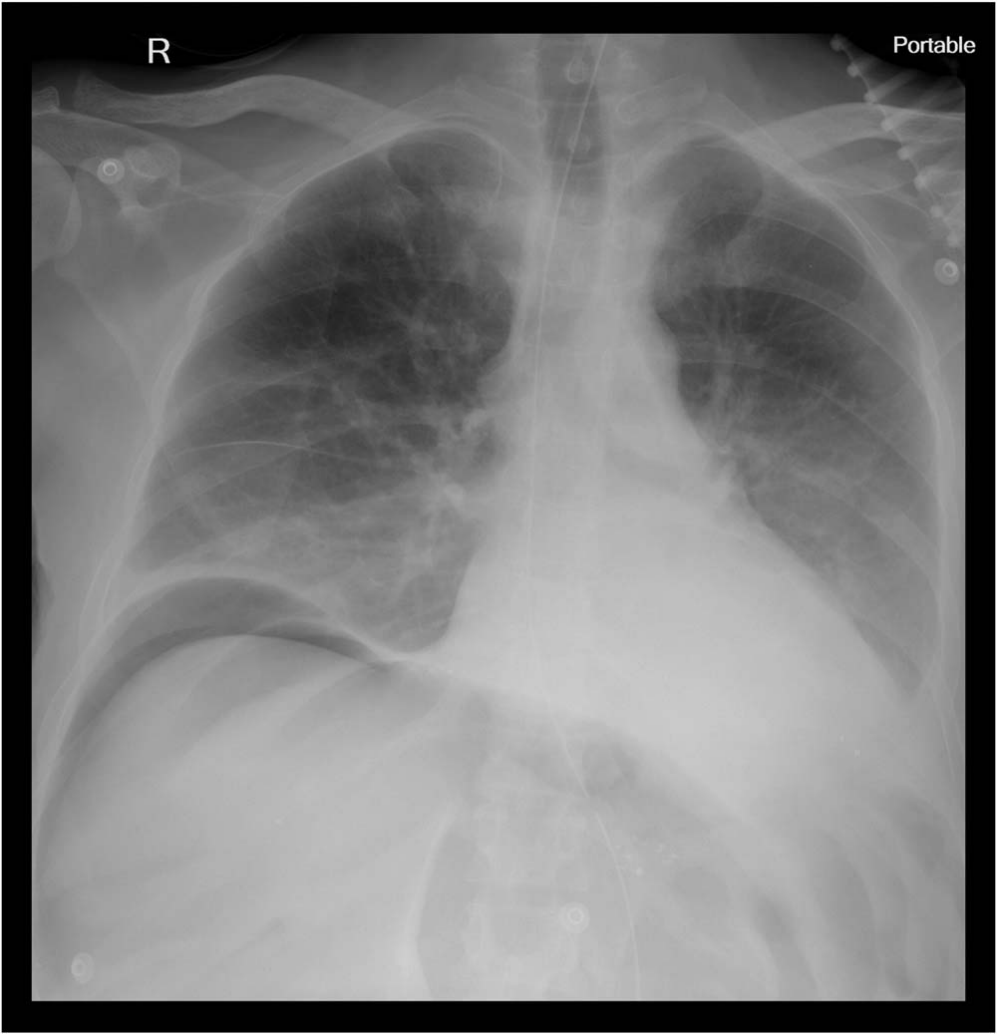
A portable chest X-ray with free air under diaphragm.

**Figure 2:**
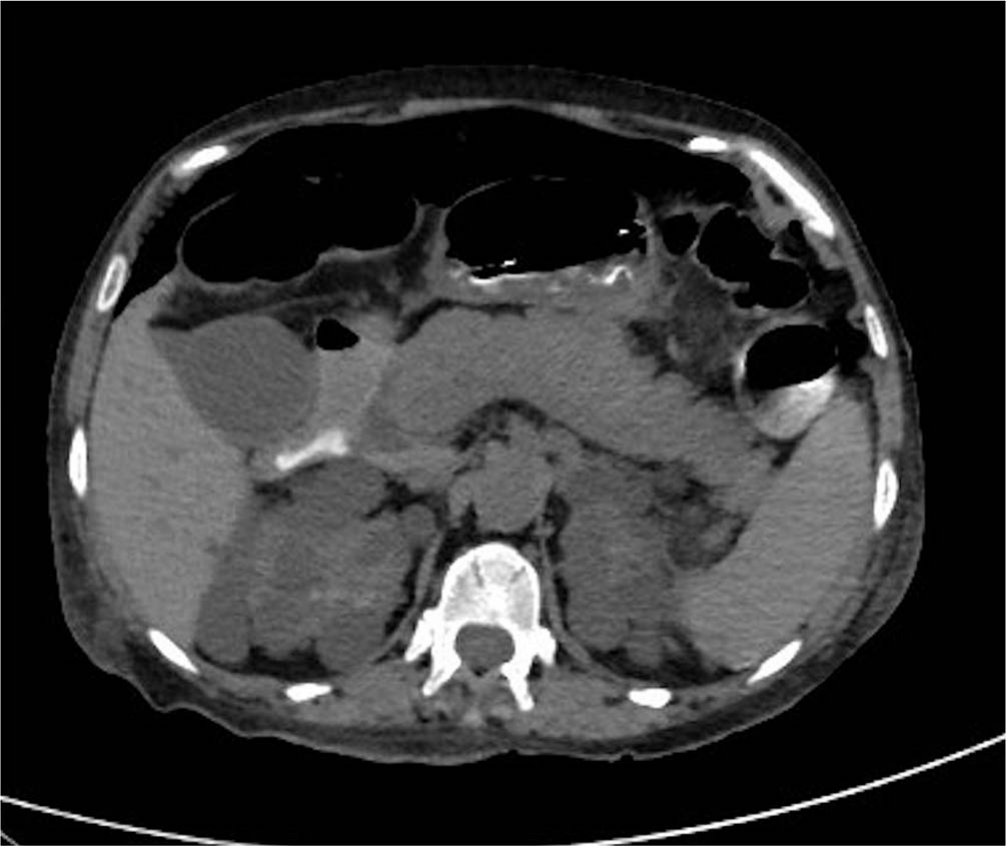
CT scan of abdomen and pelvis: pneumoperitoneum and polycystic disease in the kidneys, liver, and pancreases.

Chest X-ray revealed pneumoperitoneum (Fig. 1), and computed tomography (CT) scan confirmed free intraperitoneal air with no evident abscess (Fig. 2). The patient was admitted to the intensive care unit, resuscitated, and underwent urgent exploratory laparotomy.

### Surgical findings and intervention

**Figure 3:**
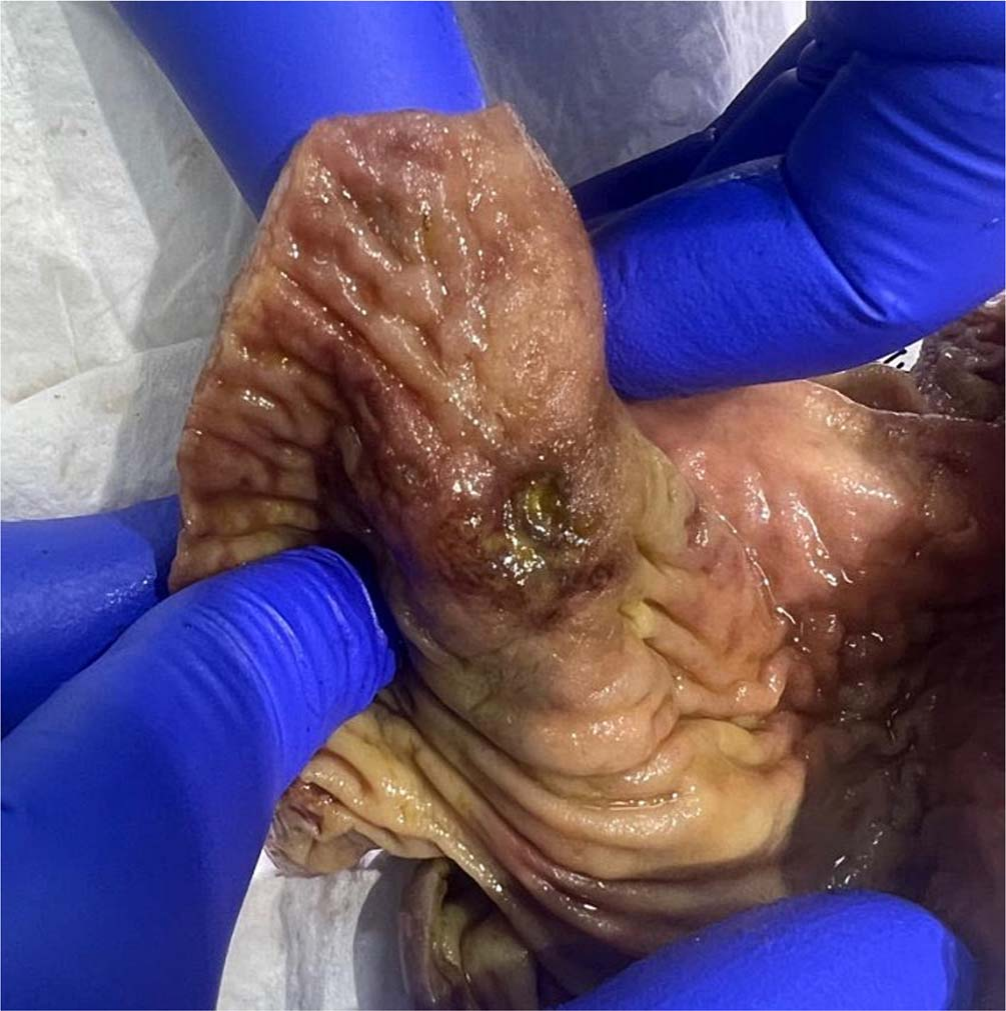
Gross pathology: the cecum shows one perforated ulcer (arrow) and one superficial ulcer.

Laparotomy revealed free intraperitoneal air and cecal perforation with two ulcers (Fig. 3). The appendix was unremarkable. A limited ileocecal resection with primary anastomosis was performed. Multiple hepatic cysts were noted and biopsied.

### Histopathological findings

**Figure 4:**
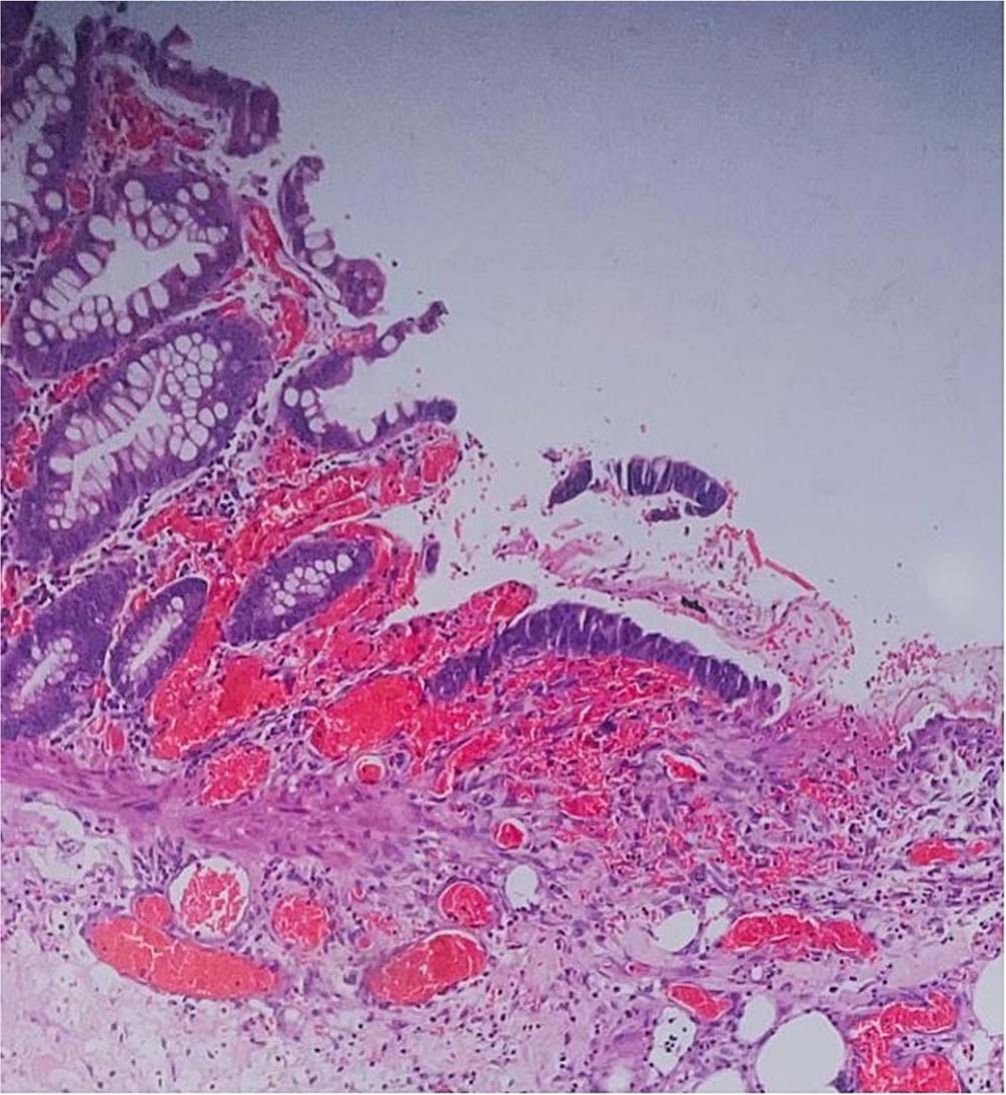
Histopathology: surface ulceration with perforation as indicated by fat lobules (arrow).

**Figure 5:**
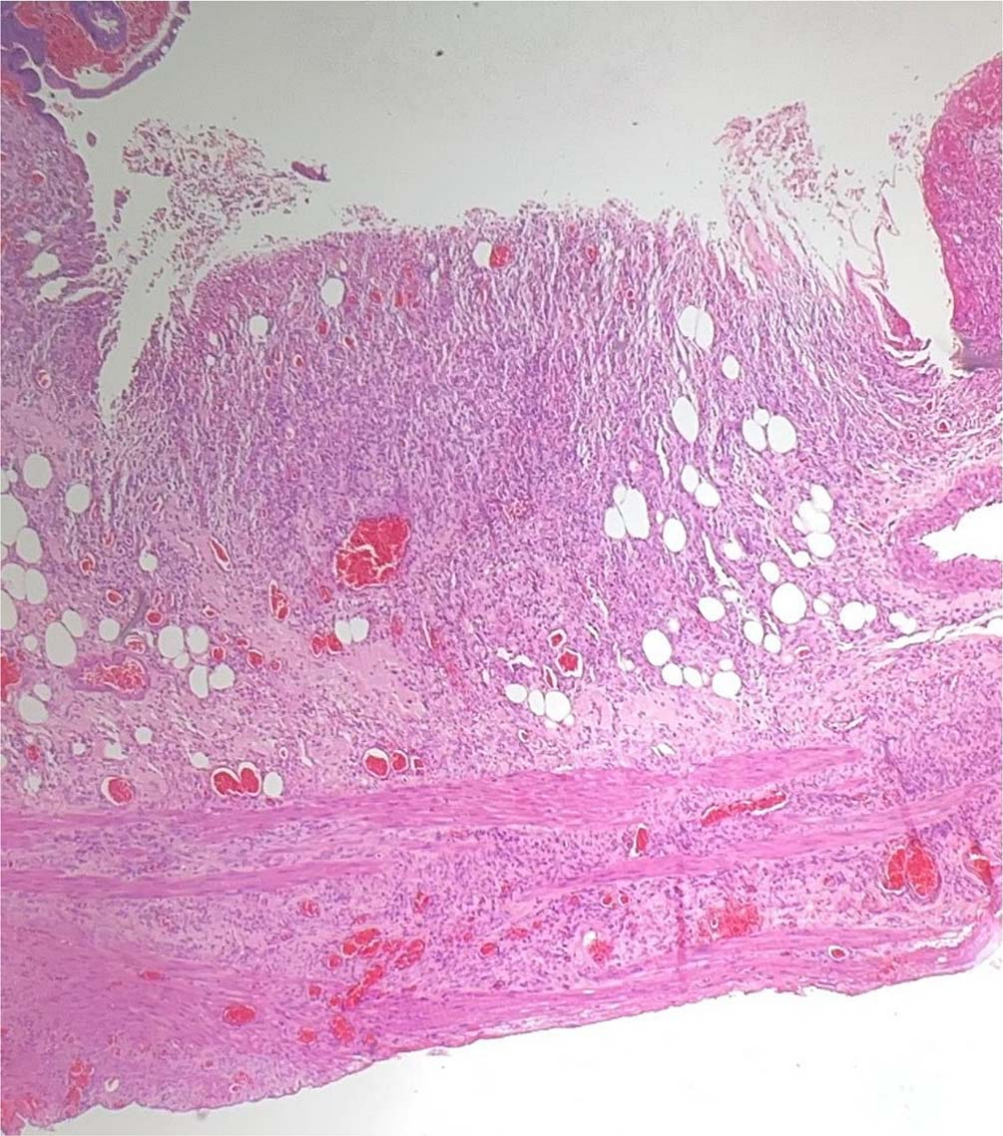
Histopathology: complete surface ulceration with granulation tissue (H&E, × 40).

Histological evaluation confirmed acute necrotizing colitis with transmural inflammation and granulation tissue (Figs 4 and 5). Hepatic cyst biopsies revealed benign biliary duct cysts with cystically dilated bile ductules.

### Postoperative course

The patient was extubated on post-operative day—1 (POD) 1, had an uneventful postoperative course, and was transferred to the medical-nephrology team by POD 5.

## Discussion

The gastrointestinal complications in ADPKD, although rare, can have severe consequences, particularly in the context of chronic kidney disease and dialysis. Dialysis-dependent patients have an increased risk of gastrointestinal pathology, including ischemic colitis and colonic perforation, likely due to chronic hypoperfusion, uremic toxin accumulation, and systemic inflammation [4, 5].

In this patient, cecal necrosis and perforation may have resulted from a combination of impaired perfusion, mucosal barrier dysfunction, and dysbiosis. Hypotension during dialysis sessions, anemia, and vascular stiffness in ADPKD contribute to colonic ischemia. Additionally, altered colonic microbiota due to uremia and frequent antibiotic exposure in dialysis patients may predispose to inflammatory damage [5].

While diverticulosis is commonly reported in ADPKD patients, particularly affecting the left colon, right-sided necrotizing colitis and perforation, as seen in this case, remain uncommon. Early diagnosis and surgical intervention are essential. Imaging modalities such as upright chest radiographs and contrast-enhanced CT scans are critical in detecting pneumoperitoneum and guiding emergency surgery.

This case emphasizes the need for heightened clinical vigilance when dialysis patients present with abdominal symptoms. Prompt imaging, aggressive resuscitation, and timely surgical exploration are pivotal to favorable outcomes.

## Conclusion

This case highlights an unusual complication of ADPKD: necrotizing colitis with cecal perforation. Clinicians should maintain a high index of suspicion in dialysis patients presenting with acute abdomen.
